# Gender Norms, Roles and Relations and Cannabis-Use Patterns: A Scoping Review

**DOI:** 10.3390/ijerph17030947

**Published:** 2020-02-04

**Authors:** Natalie Hemsing, Lorraine Greaves

**Affiliations:** 1 Centre of Excellence for Women’s Health, Vancouver, BC V6H 3N1, Canada; lgreaves@cw.bc.ca; 2 School of Population and Public Health, University of British Columbia, Vancouver, BC V6T 1Z4, Canada

**Keywords:** cannabis, gender norms, gender roles, gender relations

## Abstract

Currently, boys and men use cannabis at higher rates than girls and women, but the gender gap is narrowing. With the legalization of recreational cannabis use in Canada and in multiple US states, these trends call for urgent attention to the need to consider how gender norms, roles and relations influence patterns of cannabis use to inform health promotion and prevention responses. Based on a scoping review on sex, gender and cannabis use, this article consolidates existing evidence from the academic literature on how gender norms, roles and relations impact cannabis-use patterns. Evidence is reviewed on: adherence to dominant masculine and feminine norms and cannabis-use patterns among adolescents and young adults, and how prevailing norms can be both reinstated or reimagined through cannabis use; gendered social dynamics in cannabis-use settings; and the impact of gender roles and relations on cannabis use among young adults of diverse sexual orientations and gender identities. Findings from the review are compared and contrasted with evidence on gender norms, roles and relations in the context of alcohol and tobacco use. Recommendations for integrating gender transformative principles in health promotion and prevention responses to cannabis use are provided.

## 1. Introduction

Similar to other substances, men and boys have higher rates and frequency of cannabis use [1,2,3,4,5,6]. Boys and men also report using a greater variety of routes of administration of cannabis use compared to women and girls [7] and are more likely to use high-potency products and cannabis concentrates. These patterns of use have been linked with greater risk of developing cannabis-use dependence [8]. Young men who use cannabis are also more likely to report using alcohol and other substances, which increases the risk of adverse health and social consequences [9]. Researchers have often examined substance use from the purview of men, perceived as primarily an activity of men [10]. While the current cannabis-use patterns and trends might immediately suggest that policy and practice responses should prioritize the needs of boys and men, emerging evidence reveals the gap in cannabis-use prevalence between women and men is narrowing [11], and similar to other substances, trans and gender-diverse individuals report higher prevalence of cannabis use [12,13].

These patterns and trends in cannabis use highlight the need to attend to a range of gender-related factors. Not to be confused or conflated with sex, which refers to a range of biologically based characteristics that are linked to being male or female, gender refers to the socially constructed norms, relations, roles, expressions, behaviours and identities of girls, women, boys, men, and gender diverse people [14]. Gender is often conceptualized as a binary (e.g., woman/man). For example, masculinity and femininity have often been conceptualized in opposition to one another “as a relation of complementary difference” [15]. Yet how people understand, experience, and express gender is far more complex and varied [14]. Furthermore, as argued by Budgeon, the “gender binary which traditionally established gender hierarchy has become more multi-dimensional and complex,” (p. 318) as social norms and gender ideologies continue to change and evolve [15]. Gender norms are dynamic and embedded in the social, cultural and political context of social groups. Gender is socially constructed and individually enacted and experienced, but influenced by institutionalized power and the social, political and economic advantages and disadvantages afforded to different genders. It also intersects with other social determinants of health including social class, race, and ethnicity [16]. Therefore, studying gender in the context of cannabis use, or any other substance use, is complex, temporal and culturally specific. For further details on the features of both sex and gender as concepts, and the interaction of sex and gender in the context of cannabis use see the article published in this special issue by Greaves and Hemsing [17].

### Gender Norms, Roles and Relations

Of these multiple dimensions of gender that can be examined in the context of substance use, in this paper we focus on gender norms, roles and relations. *Gender norms* refer to societal rules and expectations that dictate the behaviors considered appropriate or desirable for people based on their gender [14]. Men and women often experience different social pressures to engage in behaviours that are reflective of traditional masculine or feminine norms. Traditional masculine norms are also sometimes referred to as hegemonic masculinity, or dominant masculinity. In some cases, extreme or strong versions of hegemonic masculinity are identifiable such as: dominance, aggression, competition, invulnerability, risk taking, stoicism, and physical and emotional control [18]. These expressions of ‘hypermasculinity’ enacted through substance use may include frequent using, binging and combining substances, all patterns which may increase the risk of negative health and social consequences. In contrast, traditional or hegemonic feminine norms include values and characteristics such as: nurturance, beauty, virtuousness and expressing emotions [19]. Dominant feminine norms tend to “emphasize risk aversion” and are typically negatively associated with substance-use behaviours in various studies [20]. The greater prevalence of substance use among boys and men may reflect differences in access to substances, with social norms affording greater permissibility for boys and men to experiment with, use substances and engage in riskier patterns of use [21].

While these dominant femininities and masculinities are archetypes, and individuals and sub-populations will deviate from them, adherence to these can be measured. The majority of research on gender norms and substance use has examined adherence to hegemonic gender norms, and particularly masculine norms. For example, the dominant masculine norms from the Conformity to Masculine Norms Inventory (CMNI) of “risk taking” and “playboy” have been strongly associated with heavy alcohol use [22,23]. Having said this, Everitt-Penhale and Ratele critique the notion of a single traditional masculinity, arguing that “traditional masculinity” varies by class, race, ethnicity and geographic context. Furthermore, they suggest that “competing traditional masculinities” are likely to exist within a single group or context [24]. In addition, Wilkinson et al. critique narrow conceptualizations of gender as either a trait (e.g., masculine personality traits) or ideology (e.g., beliefs and attitudes regarding the roles of women and men) [25]. They argue that focusing on traits lacks attention to the social construction of gender, while ideological conceptualizations narrowly focus on beliefs—one dimension of gender which does not always align with behaviors.

*Gender roles* include the expected roles and behaviours attached to the genders. Expectations about gender roles often affects and determines the opportunities available to different genders, based on culture, place and time. For example, there may be different expectations regarding substance use among girls and boys, or mothers and fathers, in different social contexts and among different cultures.

*Gender relations* refer to the interactions between genders that reflect gendered norms and affect health, behaviours and roles [14]. Femininity and masculinity can be defined both individually and relationally; for example, one’s own gender ideology may restrain substance use, while the gender norms of friends or partners, or those embedded in media may promote, or deter, substance-use behaviours [20]. Due to the social, relational and performative nature of gender and its different contexts, qualitative research is instrumental for understanding how gender norms are expressed in gender roles and relations. Therefore, investigating the relational aspects of gender is a critical area of inquiry to understand the relationship between gender and cannabis use.

While there are many cross-sectional studies and surveys analyzing gender ‘differences’ in cannabis prevalence and consumption patterns, there is limited research exploring the social factors underpinning these patterns of use. Indeed, no reviews are available on the impact of gender related factors on cannabis use. In response to this gap, we conducted a scoping review to explore the available literature on gender and cannabis use, focusing on three dimensions of gender: gender norms (societal norms regarding gender and cannabis use), gender roles (who uses cannabis and in which contexts) and gender relations (how gendered interactions influence cannabis use). In the discussion, we consider this nascent and emerging literature on gender and cannabis in light of evidence from the fields of alcohol and tobacco research and discuss opportunities for responding to various gendered aspects of cannabis use in prevention and harm reduction programming.

## 2. Methods

This scoping review on gender and cannabis is part of, and based on, a larger scoping review conducted on sex, gender and four substances: cannabis, alcohol, tobacco/nicotine and opioids.

We conducted a scoping review of the academic literature to identify, analyze and synthesize current research in: sex and gender related factors in substance use (initiation/uptake, patterns of use), effects, and prevention, treatment or harm reduction outcomes for four substances (opioids, alcohol, tobacco/nicotine and cannabis); and harm reduction, health promotion/ prevention and treatment interventions and programs that include sex, gender and gender transformative elements to address each of the four substances. A scoping review methodology was used to identify the extent of existing research on sex, gender and the four substances, and existing gaps [26]. Scoping reviews are exploratory, and unlike systematic reviews, have broad inclusion criteria and do not typically assess the quality of individual studies [27]. The scoping review was based on two broad questions:

(1) How do sex and gender related factors impact:
  (a) patterns of use;
  (b) health effects of;
  (c) and prevention/treatment/or harm reduction outcomes for opioid, alcohol, tobacco/nicotine and cannabis use?
(2) *What* harm reduction, health-promotion/prevention and treatment interventions and programs are available *that include sex, gender and gender transformative elements* and how effective are these in addressing opioid, alcohol, tobacco/ nicotine and cannabis use?

We engaged in an iterative academic literature search to identify relevant peer-reviewed studies. The searches were conducted in health-related academic databases with international coverage, including: Medline, Embase, Cochrane Database of Systematic Reviews, and Cochrane Central Register of Controlled Trials via Ovid; The Cumulative Index to Nursing and Allied Health Literature (CINAHL), PsycINFO, Social Work Abstracts, Women’s Studies International, and Lesbian, Gay, Bisexual and Transgender (LGBT) Life via EbscoHost; and Social Science Citation Index via Clarivate Analytics.

An information specialist worked with the research team to design, implement and amend the search strategy. The searches were complex, given the multiple substances and levels of intervention of interest, and various components of the concepts sex and gender. The search strategy was amended and refined based on team discussion and analysis of the search returns, articles missed by the searches, and consultation with the information specialist. The initial search covered studies published from January 2007 to August 2017, combining keywords for: sex and gender; substance use and substance-use disorders for each of the four substances (opioids, alcohol, cannabis, tobacco/nicotine); and the three levels of intervention (harm reduction, health promotion and prevention, and treatment).

After reviewing the search returns and consulting with the information specialist, the research team determined that the searches were missing key literature on the health effects of substance use (research question 1b). Therefore, the search was amended in September 2017 to include terms for health effects, and to apply additional sex and gender terms and substance-specific terms. During the process of screening returns from the second search, the research team identified multiple substance-use intervention studies relevant to the review that were not being captured by the first two searches. The information specialist analyzed the keywords used in each of the missed articles, and in April 2018 performed a third literature search with additional sex/gender terms to locate relevant studies and extend the search to cover January 2007 to April 2018. Details on the search terms used in each of these three searches are provided in Appendix A.

The three database searches resulted in *n* = 20,121 unique articles; an additional *n* = 11 records were identified through other sources. The *n* = 20,132 records were first screened by title, then by abstract and finally the full text of remaining papers was retrieved and screened a final time for inclusion. In accordance with the UK National Institute for Health and Care Excellence (NICE) manual *Methods for the Development of NICE Public Health Guidance*, abstract and full paper screening was conducted independently by two reviewers, and inter-rater reliability was compared, recorded and maintained [28]. A screening tool was used by the two reviewers to independently code the inclusion/exclusion of each study screened and the reason for exclusion. The coding decisions of the two reviewers were then compared; they participated in weekly meetings with a third researcher for the duration of abstract and full paper screening to review disagreements over the inclusion or exclusion of articles, and to resolve discrepancies by discussion and consensus.

In alignment with scoping review methods, inclusion criteria were amended post-hoc [26]. Based on increasing familiarity with the literature we used an iterative team approach to select relevant studies. The team had weekly web meetings between March 2018 and April 2019 to discuss the progress and to resolve any coding discrepancies. At the beginning of screening (February 2018) and near the end (April 2019) the team met face to face for full day meetings to discuss the scope of included literature and to further refine the inclusion and exclusion criteria. The final set of inclusion criteria, including the PICO (Population, Intervention, Comparator, Outcomes) details for framing each research question, are provided in Appendix B. Included studies were English language articles from a selection of Organization for Economic Cooperation and Development (OECD) member countries (see Appendix B for this list). The population of interest included: women, girls, men, boys, trans and gender diverse people of all ages and demographics. However, studies conducted primarily with pregnant girls and women were excluded as the research team has conducted multiple evidence reviews on substance use among this population. Studies were included that assessed: patterns of use, beliefs and perceptions regarding substance use, and health effects; and intervention studies that analyzed the impact of sex and gender or described or evaluated sex or gender informed interventions. With regard to the four specific substances of interest: tobacco and nicotine included electronic nicotine delivery systems (ENDS); alcohol use included all use and not just problematic use; opioid use included illicit and prescription opioids; and cannabis included both therapeutic and recreational use.

**Figure 1:**
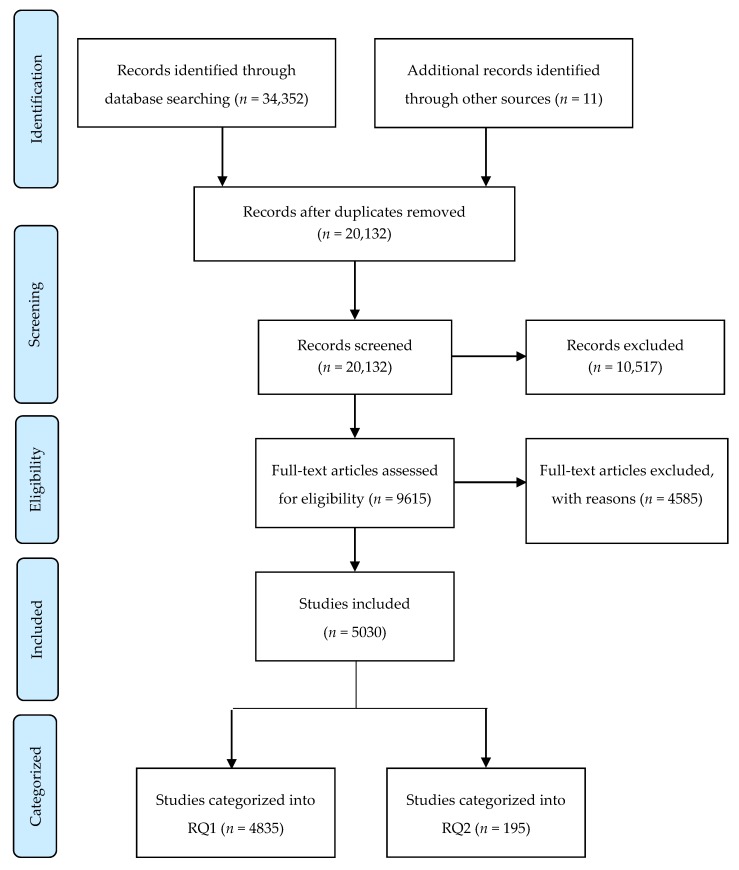
Preferred Reporting Items for Systematic Reviews and Meta-Analyses (Prisma) flow diagram. From Moher D. et al. [29].

Before acquiring papers for assessment, the *n* = 20,132 titles were initially scanned by one reviewer who removed the clearly irrelevant studies. Title screening reduced the number of included papers to *n* = 11,842. Initially, a random sample of 10% of these abstracts were independently scrutinized by two reviewers in relation to the inclusion criteria. The two reviewers achieved agreement on 83.19% of the sample of abstracts reviewed; the remaining abstracts were then divided and assessed by a single reviewer. Full papers of the remaining included studies (*n* = 9615) were then retrieved and assessed by two independent reviewers. Inter-rater reliability was monitored quarterly (each quarter of the retrieved papers) throughout the full paper screening stage to ensure the reliability score (Cohen’s kappa) remained above κ = 0.6. The final overall kappa was 0.73. After the full paper review, *n* = 5030 papers were still included (*n* = 4835 were categorized into Research Question 1 (RQ1), and *n* = 195 were categorized into Research Question 2 (RQ2)). Figure 1 provides an overview of the literature search returns, the number of papers included and excluded at each level of screening, and the final number of included papers identified.

Included studies were categorized by one researcher, who coded details on: research question (question one or two); the substance(s) addressed (cannabis, alcohol, opioids, tobacco/nicotine); primary and secondary topic (prevalence/patterns of use; beliefs/perceptions; mechanisms/biological responses; health effects or consequences; prevention intervention; brief intervention; treatment intervention; harm reduction intervention); and whether the study was a quantitative or qualitative design or a systematic review. Because the inclusion criteria were developed iteratively, and amended during screening, a second researcher checked the coding to ensure alignment with the finalized set of inclusion criteria. Once the categories were checked by a second researcher, a final searchable database of included studies was produced, with each included study categorized by substance and topic.

In total, *n* = 784 papers on cannabis were identified in the search. The majority of these papers focused on prevalence and patterns of use (*n* = 445). Additional categories included: *n* = 57 studies on interventions to address cannabis use (including prevention, harm reduction and treatment); *n* = 18 studies on beliefs and perceptions regarding cannabis use; *n* = 78 papers on biological mechanisms; and n = 186 studies on the health effects of cannabis use.

Of these, we identified *n* = 15 studies on cannabis and gender roles, norms and relations. We reviewed the reference lists of these included studies, identifying an additional *n* = 6 relevant studies. In total, we included *n* = 21 studies. Some studies were included that were conducted with one gender group if the authors explored gendered dimensions of cannabis use. While studies were excluded from the original search if they were conducted in Mexico, we chose to include these studies in this scoping review on gender and cannabis use because there were relatively few studies available examining feminine norms and cannabis use.

## 3. Findings

Details on the *n* = 21 included studies are provided in Table 1, including information on: country, study design, aims, the dimensions of gender included in the study, and key findings regarding cannabis and gender. The 21 studies included were conducted in a range of countries including: Canada, USA, Mexico, Ireland, Norway and the UK. The majority of studies were either qualitative or cross-sectional. The majority of cross sectional studies examined conformity to gender norms (e.g., based on measures of gender typicality), and qualitative studies tended to explore gender roles and relations in the context of cannabis use. A total of *n* = 8 studies included adolescents, *n* = 2 included a longitudinal design and examined cannabis use from adolescence to adulthood; and *n* = 11 included adults. See table for further details.

Findings from the studies on gender norms and cannabis use are summarized narratively in the three sections: *male typicality and cannabis use; conformity to feminine norms*; and *conformity to gender norms, culture and acculturation.* Findings from research on gender roles and relations are summarized in five sections: *reinstating and resisting dominant gender norms; cannabis and gender relations in social networks; cannabis use in intimate relationships; stigma and discrimination; and stigma among mothers and fathers who use cannabis*.

## 4. Gender Norms

### 4.1. Male Typicality and Cannabis Use

Several studies were identified measuring adherence to “male typicality” in the context of substance use, including cannabis use. Based on the theory that some boys and young men may use substances to support the development of a “male-typical or masculine” identity, Mahalik et al. explored the relationship between gender, male typicality and social norms in regards to alcohol and cannabis use, following a sample of youth from adolescence to adulthood [30]. The gender typicality measure includes 16 items assessing attitudes and behaviours demonstrated to have moderate to strong gender differences among adolescents (e.g., frequency of crying; frequency of being in serious fights) based on data from the National Longitudinal Study of Adolescent Health (Add Health). This measure identified the gender of females and males with 81.7% accuracy. Mahalik et al. applied these measures to predict the probability of each participant being male and analyzed the correlation with substance use. They hypothesized that females and males, but particularly males, who report greater conformity to male-typical behaviours and attitudes would demonstrate greater substance use during adolescence and into adulthood. Confirming their hypothesis, they found males reported greater cannabis use over time. Greater male typicality among both females and males was associated with substance use including cannabis use; however, the effect was greater for males.

Wilkinson and colleagues applied the same Add Health gender diagnostic measures, in relation to substance use from adolescence to young adulthood. However, in contrast to the study by Mahalik et al., they used multiple waves of data collection to assess gender typicality, and they assessed females and males on their adherence to female and male typicality. Similar to Mahalik et al., they also found a stronger relationship between substance use and traditional masculine gender norms for boys. Greater male typicality at wave one was associated with greater odds of high frequency cannabis and cigarette use and increased risk of use of one or more substances at wave three (during emerging adulthood). Among females, there was less change in high frequency use and polysubstance use over time. However, they caution when interpreting these findings that there is individual variability in how masculinity and femininity are understood and enacted.

### 4.2. Conformity to Feminine Norms

A systematic review examined the role of feminine norms on substance use among women. The authors were interested in individual conformity to traditional feminine norms and the relationship with substance use. The majority of studies used the Bem Sex Role Inventory (BSRI) which measures feminine traits based on societal norms, or the Conformity to Feminine Norms Inventory (CFNI) which assesses conformity to the following eight dominant feminine norms: nice in relationships, thinness, modesty, domestic, care for children, romantic relationship, sexual fidelity, and invest in appearance [19]. Their review found that 74% of studies identified a relationship between feminine norms and substance use. However, while they included search terms for cannabis/marijuana, of the *n* = 23 studies included in their review, only *n* = 2 studies included cannabis use in relation to feminine norms. All authored by Kulis et al., these studies are described in the following section.

### 4.3. Conformity to Gender Norms, Culture and Acculturation

Kulis and colleagues conducted several studies with Mexican and Mexican-American adolescents examining the impact of gender norms on cannabis use. They developed four gender constructs based on “marianismo” and “machismo”—conceptualizations of femininity and masculinity in Mexico that they argue include both positive and negative dimensions. Accordingly, the authors developed the following four constructs: *assertive masculinity* (self -confidence, personal valor and assertiveness); *affective femininity* (empathy, emotional expression, nurturing); negative masculinity or *aggressive masculinity* (a tendency to control and seek domination in relationships); and negative femininity or *submissive femininity* (dependence and submissiveness). They used 19 items to measure these four dimensions of gender identity, asking students to indicate how often they thought they exhibited gender typical traits and behaviours.

In their 2008 study, Kulis et al. surveyed adolescents in Mexico, and found that affective femininity tended to be associated with lower risks including less recent use of cannabis, while submissive femininity was not related to substance use [31]. Aggressive masculinity was associated with greater substance use including cannabis use, while assertive masculinity was only associated with perceptions of substance use among friends and receiving substance use offers. However, as the study was cross-sectional it is not possible to determine the direction of these relationships. The authors suggest that for youth identifying with aggressive masculinity, substance use may be a tool for demonstrating “toughness.” In contrast, they suggest that affective femininity may be associated with lower risk of substance use because using substances may be incompatible with aspects of this construct, such as gentleness and showing attention to others. Furthermore, they suggest that the lack of relationship of assertive masculinity and submissive femininity with substance use may reflect cultural differences between the USA and Mexico. While the USA has a more individualistic culture, in which substance use may relate to measures of assertiveness, Mexico tends to be a more collectivistic society. Similarly, they explain that submissive femininity is more strongly valued and prescribed in Mexico than the USA, and therefore boys and girls who conform to submissive femininity may not experience the same pressures to use some substances (as has been observed in studies conducted in the USA).

Two additional studies led by Kulis et al. used the same measures but with samples of Mexican-American adolescents. In one study, submissive femininity was significantly associated with alcohol use, but no significant association was found for cannabis use [32]. In a second study, they reported the following correlations regarding gender and cannabis use: assertive masculinity (assertive, self-confident, problem-solving) was associated with higher cannabis amount and frequency in girls; while assertive femininity was associated with lower levels of cannabis use in boys. Furthermore, acculturation was largely unrelated to substance use, except for cannabis use in girls [33]; highly acculturated girls who reported high aggressive masculinity (aggressive, controlling) reported the highest cannabis use. They suggest that as adolescent girls became acculturated, they may adopt certain dominant masculine norms that confer greater risk for substance use. According to the authors, *marianismo* (a Mexican conceptualization of traditional femininity) may be protective by limiting social interactions outside controlled family settings, but this protective effect may decrease with acculturation. Another explanation they offer is that as girls become more acculturated, they may be more vulnerable to using cannabis to cope with stress.

## 5. Gender Roles, Norms and Relations

### 5.1. Reinstating and Resisting Dominant Gender Norms

Several qualitative studies have explored gender roles, norms and gender relations in the context of cannabis. The performative aspect of gender expresses itself in norms of use, and through the adoption of gendered roles in relation to substance use. There is evidence that adolescents and adults “do gender” through cannabis use, and dominant femininities and masculinities can be both reinstated or resisted through cannabis use [34]. For example, in a Canadian qualitative study, adolescents were hesitant to discuss their cannabis-use behaviours as shaped by gender even though the narratives of adolescents revealed gendered social dynamics in cannabis-use settings and patterns of use [35]. For example, habitual use by girls was described as inappropriate, and girls who did smoke cannabis were often perceived as acting too “silly” and “giggly” when high, while boys who used cannabis regularly were seen as cool and relaxed. Similarly, in the qualitative study by Dahl et al., female cannabis users “did gender” in multiple ways. Predominantly, they “did traditional femininity” by not buying cannabis, remaining in control when using, smoking less and admitting when they felt anxious or too high [34]. However, some participants “did masculinity” by supplying cannabis, rolling joints, being able to consume large amounts, and enjoying being high. In contrast, men were more engaged with dealers and cultivators, often used cannabis with other men, were more likely to maximize their intoxication (e.g., by method or quantity of use) and were more open with their use.

Cannabis may also be used in ways contrary to dominant gender norms as a “way to revise or undermine gender norms” [35] (p. 2035). In the study with Canadian adolescents, boys suggested cannabis use may be associated with more androgynous values, and may represent an alternative and gentler way of “doing masculinity,” in comparison to other substance use [35]. For example, some boys described their preference for using cannabis over alcohol because it is a “happy drug” and allowed them to talk honestly and be open with their emotions; in contrast, boys explained that alcohol use among groups of boys often resulted in aggressive behavior and fights. Similarly, in a study conducted in Norway, Dahl and colleagues suggest that the “masculinity embedding cannabis use” combined two ideologies. One is a form of traditional masculinity, which tends to foster substance use, violence and sexism, and the other is a form of masculinity that “combines an ideology of gender equality with relaxation, play, fun and not taking things too seriously” [34] (p. 708). For example, men were accepting and often applauding of women who engaged in cannabis-use patterns perceived as masculine (e.g., using frequently, enjoying the high), yet they also described these behaviours as “manly” or unfeminine.

These studies from Canada [35] and Norway [34] both reveal how female cannabis users can resist dominant feminine ideals, positioning themselves as “one of the boys” by engaging in cannabis-use activities traditionally identified as more masculine. Similarly, a qualitative study by Arnull and Ryder described alcohol and cannabis use among a sample of justice-involved girls in the UK and USA as a way of “doing gender control” by resisting “hegemonic norms [framing]…[alcohol or drug] use as unusual, unfeminine or non-agentic” [36] (p. 1365). By sharing the girls’ narratives, they argue that substance use among girls is both a “pleasurable and boundaried” activity for girls. The authors stress the role of girls as agents in making decisions regarding their alcohol and cannabis use, rather than framing girls’ substance use as deviant, “unfeminine” or caused by trauma.

### 5.2. Cannabis and Gender Relations in Social Networks

Qualitative research reveals gendered social dynamics in accessing cannabis. Hathaway et al. conducted interviews with social sciences students attending universities in Ontario and Alberta regarding their substance use [37]. Young women who used cannabis discussed the benefits of gaining access to cannabis via their male friends. As one young woman said:

*“I have never really bought it. I always sort of smoke other people’s weed. Like I have this friend of mine. He is a really nice guy, and I usually smoke with him and his friends. They never let me pay, because they say I don’t smoke much…but I really think it’s because I am a girl and they are trying to be nice (laughs) (Female, 18).”* [37] (p. 1675).

The authors suggest that buying and maintaining a supply of cannabis is typically a male activity, but that some women may access cannabis for free through their relationships with men. Similarly, a qualitative study with Canadian adolescents found that among some participants, girls were perceived (by both girls and boys) as more easily accessing cannabis [35]. While men are usually the dealers or suppliers, girls were described as flirting and using their beauty or “sexuality as a tool” to access cannabis for free. As one male participant explained:

*“Because a lot of the dealers are men and women have a lot of power of persuasion over men, especially if they are beautiful women. It’s easy for them to get what they want out of men, so there’s a bit of manipulation that goes on there”* (p. 2034).

There are also gendered social dynamics regarding cannabis use among male friend groups. A qualitative study explored men’s greater prevalence of illicit psychoactive substance use in Ireland in relation to masculinities [38]. Darcy argues that men use illicit substances to navigate masculinities in “paradoxical ways.” They found that some of the men’s substance-using behaviours aligned with hegemonic masculine ideals–including notions of “toughness,” competition and endurance of physical and emotional strain. For example, they described “competitive drug taking” scenarios in which experienced cannabis users would consume cannabis using a combination of methods (e.g., bucket bongs, gravity bongs), with the purpose of *“seeing, how high, how far, how fast. Last man standing, whatever it might be”* [38] (p. 11).

Certain ways of consuming cannabis, including the methods used, the intensity, and the combination with other substances may provide opportunities for men to demonstrate their masculinity by showing the control they have over their bodies. The authors argue this is a form of gender performance. However, other ways of performing masculinity, or resisting dominant masculine norms, emerged. For example, men described how using cannabis facilitated closeness and allowed men to express their emotions; in particular, among heterosexual men, cannabis allowed opportunities for men to “contravene conventional gender expectations” regarding expressing emotions and openness between male friends [38].

In a second paper based on qualitative data collected with the same sample of men, the offering and sharing of cannabis with other men was perceived as a sign of friendship [39]. While using cannabis together was described as a “social leveler,” possessing and providing cannabis to other men was identified as facilitating an elevated social position and changing the social dynamic. In addition to providing a space where men could perform traditional masculinity via cannabis use (achieving dominance by obtaining and supplying substances including cannabis), cannabis use provided opportunities for bonding with male friends and being more emotionally expressive. Similarly, in an ethnographic study with low income, criminally involved young men living in Ireland, buying, maintaining and consuming cannabis strengthened social bonds with other men, with them consuming cannabis together in “a regularity that approached ritual” [40] (p. 8).

One study explored substance use, including cannabis use, in the context of girls’ friendships. Arnull and Ryder argue that public health approaches have focused narrowly on the risks of substance use, avoiding both the pleasurable functions of substance use, and the efforts of people who use substances to manage and minimize risks. By sharing the voices of a group of justice-involved girls, they describe how girls negotiate risks and use substances for social bonding and pleasure. Girls reported having fun with friends while using substances and experiencing pleasure from intoxication. They also described how they relied on their friend group to prevent or reduce physical and sexual risks of alcohol and cannabis use. For example, girls discussed remaining with their girlfriends when they went out partying, ensuring their friends arrived home safe or staying in each other’s homes if they were too intoxicated.

### 5.3. Cannabis Use in Intimate Relationships

There is evidence from qualitative research on cannabis use and gender relations in intimate heterosexual relationships. In a Norwegian study conducted with people who had reduced or quit using cannabis, some participants discussed changing their cannabis-use patterns to please a partner. This theme was central in interviews with young men, but only one woman discussed stopping her daily cannabis use when she began a new relationship with a man who did not use cannabis [41]. Some men described engaging in arguments and conflicts with their partners regarding reducing or quitting cannabis use, while others described their change in use as unproblematic. For example, one man in the study described quitting cannabis when he moved in with his non cannabis-using partner, explaining: “*it would be sort of excluding if I was to be on a different mental level*” (p. 180). Men negotiated the frequency, occasion and context of their cannabis use to please their partners, and several described this shift as a natural progression from youth to adulthood. However, the authors caution that the findings from this study may have limited generalizability as participants were relatively socially advantaged with 19 of the 25 men having a higher education. These findings may not be translatable to cannabis users who are experiencing social disadvantage.

In one qualitative study examining substance use and sexual experiences among young adults, alcohol was commonly used by young men for pursuing potential sexual partners, and young women reported being more accepting of sexual offers from men when using alcohol [42]. In contrast, when using cannabis, young women reported being more selective regarding sexual partners. Both young women and men reported feeling more in control on cannabis than alcohol, but also quieter and less social. Women and men who used cannabis prior to a sexual experience reported greater post-sexual satisfaction compared to those who used alcohol before sex, while participants who drank alcohol reported greater regret following sex. Some participants reported that the illegal nature of cannabis occasionally meant more private use that sometimes facilitated sexual encounters [42].

Similarly, in a qualitative study with cannabis users and retailers in Florida, some men discussed the role of cannabis for facilitating private moments with women they were attracted to [43]:

*‘Kara is the one that I’m quite fond of, she smokes in my bathroom at all the parties… So being able to steal Kara was very easy to do with just [saying to her] “Hey why don’t you come and have a conversation with me in my bathroom?”* (Matthew, age 30) (p. 761).

While these gender relations have been observed in the context of illegal cannabis markets, the hidden nature of use and opportunities for privacy may diminish as cannabis use becomes legal, openly consumed, and socially normalized [42]. Nonetheless, it seems clear that the role of cannabis in intimate heterosexual relationships may be somewhat different than that of alcohol.

### 5.4. Stigma and Discrimination

There is a lack of research examining the impact of gender roles and relations on cannabis use among people with a range of sexual orientations or diverse gender identities. Yet, several studies suggest cannabis may be used to cope with experiences of stigma and discrimination related to not conforming to predominant gender norms and roles. In a qualitative study on the impact of anxiety on cannabis use among bisexual women in Canada [44], some women described experiencing a lack of belonging, and how this contributed to using cannabis to manage anxiety. The authors suggest that cannabis may be used as a way to cope with not conforming to gender roles, or the stress related to experiencing multiple forms of oppression and discrimination related to being a bisexual woman, including sexism and biphobia. For women who experience these social disadvantages, cannabis may be used as a way to facilitate social belonging. This is also elucidated in their findings from an earlier study in which cannabis use was correlated with higher levels of social support among bisexual women, and described during focus groups as a tool for social connection [45].

Gender identity has also been examined in a study examining the relationship between gender minority stress and substance use among transgender women and men in the USA where the authors found that transgender men reported higher rates of cannabis use compared to transgender women. The authors note that this is similar to findings among general populations of women and men who do not identify as transgender, suggesting that gender socialization may also influence cannabis use among transgender people. Gender dysphoria, defined as the conflict between one’s sex assigned at birth and gender identity, was associated with cannabis use among both transgender women and men. Additionally, among transgender women gender minority stress was associated with cannabis use [46]. The authors conclude that transgender individuals may use cannabis to “validate and affirm their gender identities” and identify the need for more research to explore the differences in cannabis use among transgender men and women.

### 5.5. Stigma among Mothers and Fathers Who Use Cannabis

Substance use tends to be perceived as more socially acceptable for men than women. In particular, gender norms that position women as mothers and caretakers are defined in opposition to substance use. Women who are mothers have identified stigma associated with cannabis use [34]. Women often report stopping cannabis use when they transition to motherhood because of this stigma, and those who do not report experiencing social disapproval [47]. Dahl argues that women experience more social controls at an earlier age compared to men [41]. In a qualitative analysis of cannabis use and stigma among a sample of cannabis users in Canada, Hathaway and colleagues discuss how stopping substance use during pregnancy was expected among women [47]. Women who smoked cannabis during pregnancy reported experiencing social disapproval [47]; as one woman remarked:

*“When I was pregnant, I had morning sickness all day, every day for nine months, but I smoked only a few times. There was a strong social stigma against me. People told me not to smoke. (Paralegal, 41)”* (p. 462).

Women who were parents of adolescents described being afraid of child welfare involvement and feeling hypocritical if they were using and hiding their cannabis use from their children. In order to manage this, women limited where and when they used cannabis to avoid having their children and others knowing about it. The authors describe the women as internalizing stigma regarding their cannabis use and engaging in practices of “moral regulation” to maintain their mothering role, as well as others’ perception of them as a “good mother.”

Similarly, in a qualitative study with parents of children who had used cannabis, participants revealed normative gender roles and the expectations that women experience [48]. Mothers described feeling like failures if they had experienced challenges regarding their child’s substance use, and often encountered a lack of social support due to the judgement and stigma. While this appeared to be more salient for women, one father also expressed feeling judgement and stigma regarding asking for help with parenting challenges related to substance use. Furthermore, the authors argue that focusing on the parent-child unit as the site for preventing and responding to substance use is problematic because it individualizes substance use and decontextualizes it from the influence of social factors.

One study found that men also perceived cannabis use as incompatible to their role as fathers. In a qualitative study conducted with people who had reduced or quit using cannabis in Norway, participants who were parents or who were expecting a child discussed cannabis use as being incompatible with parenting, particularly due to fear over the consequences of using an illicit substance [41]. One father said it would be “out of the question” to keep cannabis in the home, and multiple men spoke of the dangers of buying and using cannabis in the context of fatherhood. As one man explained:

*“Smoking hash isn’t that dangerous, but being caught and stigmatized as a criminal—a criminal parent of young children; is that what I am? That is quite a poor starting point for being a family man, as you’re supposed to be”* (p. 178).

Men who were expectant fathers also discussed reducing or stopping to support the transition in their role to fatherhood. Some men qualified that they do not perceive cannabis use during parenting as necessarily harmful, but with the new responsibility of caring for and protecting their child, they felt uncomfortable with the idea of using cannabis while parenting. However, some men did convey a sense of loss with the shift in identity from cannabis user to a non-using father.

## 6. Discussion

Based on limited, but emerging evidence, it is clear that gender norms, roles and relations impact patterns of cannabis use in a range of ways. Several correlational studies examined the relationship between adherence to gender norms and cannabis use, reporting an association between measures of masculinity (specifically, male typicality) and cannabis use [20,30]. Most research on adherence to dominant masculine norms or male typicality and health behaviours has reported a negative effect on measures of health, including higher rates of substance use and dependence [22]. However, the relationship between gender norms and behaviours, including those surrounding substance use, is complex. Some masculine norms may actually be associated with health promoting behaviours. For example, the “winning” and “competition” subscales of the CMNI have been associated with protection from substance use and misuse, and may have application in promoting health among men [49].

Studies examining the relationship of adherence to feminine norms with cannabis use are lacking. However, similar to the research on masculine norms and substance use, there is evidence that some feminine norms may be protective of substance use while others may increase risk. For example, women who conformed to traditional feminine norms identified in the CFNI, including “sexual fidelity” and “modesty,” have reported lower likelihood to engage in binge drinking. However, the feminine norm “relational” was associated with increased binge drinking [50]. Adherence to some ‘masculine norms’ by young women is also associated with substance use [20]. For example, a study with college women in the US found that female adherence to certain masculine norms (as identified in the CMNI), including ‘risk-taking” and “emotional control,” was associated with binge drinking [50]. Further research is needed to examine the relationship of specific traditional feminine or masculine norms with cannabis use and how they operate across genders.

Studies measuring adherence to gender norms have been critiqued for underestimating the complexity of the relationship of gender norms with various social factors including race, ethnicity, religious identity, and sexual orientation [22]. For example, cross-sectional study designs assessing measures of male typicality or adherence to masculine or feminine norms at specific time-points may erroneously imply that gender norms are fixed [19]. For example, the CMNI and CFNI do not recognize or integrate historical or developmental changes in gender norms or the influence of culture and social and political contexts [20]. Additionally, Wilkinson et al. argue that gender ideologies and the expression of gender norms changes with age, especially during transitional periods from adolescence to adulthood that involve changing relationships, roles, employment, social settings and responsibilities [20]. Recently, there has been much greater understanding of gender as fluid and socially constructed.

Indeed, gender is both socially constructed and individually enacted, and traditional masculinities and femininities can be both reinstated and reimagined through cannabis use. In addition to discussing how adherence to traditional gender norms influences substance use, findings from several qualitative studies show how substances may be used to challenge or disrupt societal gender norms. As described by Robertson and colleagues, masculinities are complex, dynamic, and can be expressed in diverse ways [51].

Research on alcohol use and tobacco use among girls and young women has also explored how substances may be used to transcend and contest certain femininities. For example, a study conducted in Spain describes how female adolescents use alcohol in public spaces as a way of challenging social expectations regarding femininity that have typically restricted their use of public space and substances [52]. Similarly, qualitative research reveals that young women can frame their alcohol [53] and tobacco use [54] as a form of rebellion against traditional gender roles. These complex and sometimes contradictory ascribed meanings of tobacco use can persist into adulthood among women [55,56]. As social norms and gender ideologies continue to evolve [15], further research is needed to examine how gender norms are perceived, expressed and contested, how these meanings persist or change through the life cycle, how they may differ across cultures, and how this influences cannabis-use patterns.

While cannabis use is becoming more socially acceptable, findings from the review suggest that stigma remains high among pregnant women and mothers who use cannabis. This is also true for other forms of substance use. Among women, substance use is considered in conflict with traditional feminine norms and gender roles. Women who use substances during pregnancy and parenting are often perceived as selfish and uncaring, and in opposition to the traditional role of the “good mother” [57]. Applying a feminist embodiment approach to substance use, Ettorre discusses how substance use among women tends to focus narrowly on the health effects for the fetus, with women’s bodies reduced to “fetal containers” [58]. Women who use substances are perceived as “unfit to reproduce”, and pregnant women who use substances are perceived as “lethal fetal containers” [58]. She stresses the importance of addressing stigma and discrimination and maintaining the basic human right of reproductive choices regardless of substance use. Indeed, it is important to see women’s health and substance use as in itself worthy of harm reduction-oriented support, whether during pregnancy or motherhood, or in general. Service approaches that consider three clients as important: the mother, the child, and the mother-child unit are increasingly being advocated [59].

The Norwegian study conducted with women and men who had recently reduced or stopped smoking cannabis found that men described fatherhood as incompatible with cannabis use, although this was discussed largely in the context of fear of legal consequences (in Norway, where cannabis is an illegal substance) rather than social disapproval [41]. Men also described reducing or quitting using cannabis if their partner disapproved of their use, although some men did cite arguments and conflicts. Similarly, researchers in Canada have explored the experiences of fathers who smoke [60] and developed and evaluated gender-sensitive resources for men [61]. In a qualitative study on men’s experiences of quitting during the transition to fathering, they found that men often experienced disapproval from their partners and sought to maintain their autonomy while experiencing pressures to stop smoking [49]. Further research is needed to identify opportunities for addressing gender norms in cannabis use in harm reduction and health promotion efforts.

There is a general lack of research on gender norms, roles, relations and cannabis use among non-heterosexual people and people with diverse gender identities. Yet trans and gender-diverse youth report high rates of substance use, mental health issues and violence and trauma, and transgender women and non-binary assigned male at birth youth tend to report greater substance use [62]. Similarly, among young adults, high rates of tobacco use have been reported among both sexual minority females and gender minorities [63]. Further research is needed to understand how substance use among trans and gender diverse people, and cannabis use in particular, is shaped by gender norms, roles and relations. In addition, qualitative research on the experiences related to gender and cannabis use among people of diverse sexual orientations is needed to explore the complex relationships between sexual minority status, heterosexuality and gender roles and norms. Existing evidence highlights the need for integrating social supports in responses to prevent and address cannabis use among both groups: people of non-heterosexual orientations and diverse gender identities.

More research is also required to explore how gender intersects with other social determinants of health to influence cannabis use. In our review, we found several studies exploring the relationship between gender and culture or acculturation and substance use [31]. Some qualitative studies included sub-groups of males or females experiencing social disadvantage, including: low income men [40], and justice involved girls [36]; however, these studies did not analyze the intersection of gender and social disadvantage in relation to cannabis use. Yet evidence from the wider substance use field suggests that other social dimensions of health influence how we act, respond to, or “do gender.” For example, in an intersectional analysis of women’s smoking, the authors contend that the ability to challenge traditional social constructions of femininity is typically a privilege reserved for women belonging to higher social class [54]. More nuanced research is required to explore how other social determinants of health intersect with gender to shape cannabis-use experiences.

In summary, addressing gender norms, roles and relations in health-promotion messaging regarding cannabis use is critically important. Evidence from the review suggests that these dimensions of gender can have an effect on harms, risk and exposure. As more evidence emerges on gender and cannabis use, it is critical to avoid approaches to either prevention or health promotion that are gender exploitive and reinforce negative gender stereotypes. For example, an analysis of substance use education in Australia describes how school-based substance-use education reproduces harmful feminine and masculine norms by framing young women’s substance use as more problematic than men’s and blaming women for the physical and sexual victimization they are at risk of while intoxicated [64].

But advancing beyond approaches that merely avoid harmful gender stereotypes, health-promotion responses are needed that actively integrate gender transformative principles. Rather than just reflecting gender-based factors and concerns in messaging, gender transformative health promotion is aimed at improving gender equity *at the same time* as improving health [65]. Evidence from this review suggests there may be substantial opportunities for both gender-responsive and gender-transformative responses to cannabis use. For example, messaging might address shared responsibility for abstinence from cannabis use during pregnancy and parenting, and resources and supports could be developed for men to reduce or quit cannabis use during pregnancy and parenting, emphasizing the role of men as providers and protectors, similar to smoking cessation resources that have been developed for men [61]. In addition, messaging could address gendered risky patterns of use, including: cannabis and alcohol use, competitive use among men, and driving and riding as a passenger after cannabis use. Finally, there is a need for stigma reduction among pregnant women and mothers and fathers who use cannabis. One way stigma can be reduced is by providing accurate information regarding the health effects of cannabis use during pregnancy and parenting, while avoiding language that is judgmental and shaming.

## 7. Conclusions

While research on gender and cannabis is in its infancy, the available literature indicates that, similar to other substance use, gender norms, roles and relations have the potential to strongly influence patterns of cannabis use. How gender is expressed through cannabis use is complex, culturally specific, multi-faceted, and ever-evolving. As gender norms, roles and relations are constantly in flux, ongoing research is needed to explore the relationship between gender and cannabis use that is situated in the social, cultural and political context. Further research is also needed to understand how people belonging to diverse gender identities perceive and express gender through cannabis use; and that investigates how gender intersects with other social determinants of health including: sexual orientation, class, race and ethnicity. Harm-reduction, health-promotion and prevention messaging approaches are needed that address substance use and gender norms, as well as structural and institutional factors that specifically support harmful gender norms and behaviours. Specifically, gender transformative principles can be integrated in prevention, harm-reduction and health-promotion messaging to advance gender and health equity simultaneously, and erode the impact of negative gender stereotypes and stigmas. All of these gender-related issues need to be visited as cannabis use becomes more regulated, decriminalized or legalized in various jurisdictions around the world.

## Figures and Tables

**Table 1:** Study details.

Author/Year	Country	Study Design	Study Aim	Population	Assessment of Cannabis Use	Dimension of Gender Addressed	Gender and Cannabis Findings
Arnull and Ryder 2019	UK and USA	qualitative comparative study	To prioritize the voices of justice-involved girls in the UK and USA regarding their reasons for substance use	age 13–18 adjudicated girls who had been sentenced for a violent offense; *n* = 24 girls in USA (primarily identified as “women of color”), *n* = 35 in UK (primarily White British)	Participants were assessed for eligibility based on self-reported “ever use” of cannabis and alcohol	Gender relations; explored use of alcohol and cannabis, within justice involved girls’ social groups.	Girls described pleasure related to their cannabis use with other girls. Within their friend groups they managed physical and sexual risks when using substances.
Belackova and Vaccaro 2013	USA	qualitative	To explore the role of cannabis in friendship groups	*n* = 44 adult cannabis users and retailers in Florida; *n* = 32 men and *n* = 12 women; primarily White	Participants were assessed for eligibility based on self-reported use of cannabis in past 12 months	Gender relations in the context of reasons for/functions of cannabis use.	Some men described opportunities for pursuing intimate interactions with women when using cannabis.
Brady et al. 2016	USA	systematic review	To examine feminine norms and substance use outcomes among women	only *n* = 2 studies included cannabis use (Kulis 2008; and Kulis 2010, see below)	Not reported	Gender norms; studies were eligible for inclusion if examined feminine norms/ideology or feminine role conflict.	Majority of studies reported that adherence to feminine norms increased substance use, but only two studies included cannabis (included below).
Dahl and Sandberg 2014	Norway	qualitative	To examine how women navigate a gendered cannabis-use culture in Norway	Analyzed data from 2 studies: one with *n* = 100 cannabis using adults; and one with *n* = 25 experienced cannabis users (*n* = 7 women; *n* = 18 men)	Participants were assessed for eligibility based on self-reported long term cannabis use; included sporadic to heavy use (not quantified)	How adults “do gender” through cannabis use; examined women and men’s roles and positions in social networks using cannabis, and their concerns about use.	Dominant femininities and masculinities are both reinstated and reimagined through cannabis use.
Dahl 2015	Norway	qualitative	To examine the change in identity among experienced cannabis users who had quit or reduced their use	*n* = 7 women, *n* = 18 men; Age = 23–40 years; former daily cannabis users who had reduced or quit using cannabis without formal drug and alcohol treatment	Participants were assessed for eligibility based on self-reported former daily cannabis use	Gender roles and gender relations in the context of reducing and quitting cannabis use.	New fathers discussed the cannabis user identity as incompatible with their role as father; men discussed changing their use in the context of intimate relationships.
Darcy 2019	Ireland	qualitative	To explore how men’s illicit substance use patterns and intoxication converge with masculinities	*n* = 20 Irish men who used illicit substances (*n* = 17 heterosexual; 2 homosexual; 1 undeclared)	Participants identified as “recreational illicit drug users”	Gender relations; gender norms; applies a gender lens to examine Irish men’s illicit substance using practices in the context of masculinities, and within the context of use with other men.	Men use illicit substances as a way to navigate traditional masculinity in paradoxical ways: both for closeness in friendships, and in competition.
Darcy 2018	Ireland	qualitative	To explore men’s substance use as a friendship practice	Same as above	Participants identified as “recreational illicit drug users”	Gender roles and relations; how cannabis is used in friendships and social settings, and in relation to conventional masculine stereotypes.	Cannabis use provided opportunities to “contravene conventional masculine stereotypes” (e.g., by offering a space for bonding with male friends, being more emotionally expressive), as well as reinforced masculine stereotypes (e.g., expressing dominance by obtaining and supplying substances, including cannabis).
Gonzalez, Gallego, and Bockting 2017	USA	cross-sectional	To examine the relationship between gender minority stress and substance use among transgender adults	*n* = 1210 transgender adults (*n* = 680 transgender women; *n* = 530 transgender men)	Participants were asked: ‘‘In the last three months, how many days did you use marijuana or hashish (weed, grass, reefers)?’’	Gender roles (non-conformity, gender minority stress), gender dysphoria and cannabis use.	Gender dysphoria was associated with cannabis use among both both transgender women and men; among transgender women, gender minority stress was associated with cannabis use.
Haines-Saah et al. 2019	Canada	qualitative	To highlight the perspectives of parents on preventing problematic adolescent cannabis use, and critique notion of ‘parents as the best prevention’	*n* = 16 parents of children (over age 13) who used cannabis; mostly women (*n* = 12)	Participants were eligible to participate if they were a parent of a child over age 13 who had experience with cannabis use	Discusses gender roles: expectations of mothers.	Mothers described feeling like failures if they had challenges regarding their child’s substance use, and experienced a lack of social support due to judgement and stigma.
Haines et al. 2009	Canada	qualitative	To explore how adolescents perceive cannabis-use experiences as influenced by gender	*n* = 45 adolescents, 13–18 years; *n* = 26 boys, *n* = 19 girls	Participants included frequent cannabis users (minimum of past week use)	Gender norms, roles and relations; gender was coded into several sub-themes: styles of use by boys and girls; sex differences in use; gender and access; use in the context of relationships; issues of safety when smoking or ‘‘partying’’. Analysis focused on how students spoke about gender.	Girls and boys described gendered social dynamics in cannabis-use settings and patterns of use.
Hathaway et al. 2011	Canada	qualitative	To examine extra- legal forms of stigma based on interviews with cannabis users	*n* = 92 (mean age 39) who had used cannabis on 25 or more occasions	Eligibility screening survey identified participants with personal experience with cannabis i (lifetime prevalence)	Gender roles; examines stigma in the context of cannabis use and the disadvantages and benefits of using.	Women described experiencing stigma when using cannabis during pregnancy and as mothers; conflict with the role of “good mother.”
Hathaway et al. 2018	Canada	qualitative	To examine patterns of supply of cannabis among students at Canadian universities	*n* = 130 social sciences students in universities in Ontario and Alberta (55% female; 47% reported ever using cannabis)	Eligibility screening survey identified “regular” or “occasional” cannabis users (not quantified)	Gender relations in the context of cannabis supply.	Buying and maintaining a supply of cannabis was typically a male activity.
Ilan 2012	Ireland	qualitative	To explore the experience of street culture among socio-economically disadvantaged young men in Ireland	*n* = 7 adolescents and young men engaged in street culture in Dublin	Not reported	Gender relations in the context of male friendships.	Cannabis was used to facilitate male friendships, social bonding.
Kulis et al. 2008	Mexico	cross-sectional	To examine the relationship of femininity and masculinity constructs developed for Mexican-American youth with a range of substance use outcomes	*n* = 327 adolescents in Mexico	Self-report past 30 day use of cannabis (Likert scale)	Gender norms; assessed four constructs based on Mexican concepts of *marianismo* and *machismo* including: aggressive masculinity, assertive masculinity, affective femininity and submissive femininity.	Aggressive masculinity was associated with greater risk of substance use for most outcome measures, while affective femininity was generally associated with lower risks including less recent use of cannabis.
Kulis et al. 2010	USA	cross-sectional	To examine the relationship of femininity and masculinity constructs with substance use among Mexican-American youth	*n* = 151 Mexican-American adolescents	Self-report past 30 day use of cannabis (Likert scale)	Same as Kulis et al. 2008.	Submissive femininity was significantly associated with alcohol use; no significant association was found for gender role and cannabis use.
Kulis et al. 2012	USA	cross-sectional	To examine the relationship between adaptive and maladaptive constructs of masculinity and femininity, substance misuse and acculturation among Mexican-American youth	*n* = 1466 Mexican-American adolescents	Self-report past 30 day use of cannabis (Likert scale)	Same as Kulis et al. 2008.	Highly acculturated girls who reported high maladaptive masculinity (aggressive, controlling) reported the highest cannabis use.
Mahalik et al. 2015	USA	cross sectional longitudinal	To examine the relationship between gender, male-typicality, and social norms on longitudinal patterns of alcohol intoxication and cannabis use in US youth	*n* = 10,588 youth (48% male; 52% female)	Self-report days per month cannabis use (Likert scale)	Gender norms; adherence to male typical behaviours and attitudes among females and males from adolescence to adulthood (based on measure of male typicality from Add Health data).	Greater male typicality among both females and males was associated with substance use including cannabis use; however, the effect was greater for males.
Palamar et al. 2018	USA	qualitative	To examine and compare cannabis users’ psychosocial and physical sexual experiences and sexual risk behavior	*n* = 24 adults (*n* = 12 women; *n* = 12 men); all heterosexual	Participants were eligible to participate if they self-reported sexual intercourse while high on cannabis in the past 3 months	Gender relations; cannabis use in the context of heterosexual sexual relations.	Young women reported being more selective regarding sexual partners when they were using cannabis. Participants (female and male) reported feeling more in control on cannabis than alcohol, but also quieter and less social.
Robinson 2015	Canada	mixed methods	To examine the impact of anxiety on cannabis use among bisexual women	*n* = 92 bisexual women ages 18–54	Self- report cannabis use in the past year (Likert scale) using the Drug Use Disorders Identification Test-Extended Version (DUDIT- E)	Non-conformity to gender roles and impact on stress and substance use.	Cannabis may be used as a way to cope with “female gender roles”, and discrimination based on gender and sexual orientation.
Robinson, Sanches, and MacLeod 2016	Canada	correlational	To examine the prevalence and mental health correlates of illicit cannabis use among bisexual women	*n* = 262 bisexual adult women	Self- report cannabis use in the past year (Likert scale) using the Drug Use Disorders Identification Test-Extended Version (DUDIT- E)	Gender relations; non conformity to gender roles and social exclusion.	Cannabis use correlated with social support; bisexual women who often face social exclusion may use cannabis as a tool for social connection.
Wilkinson et al. 2018	USA	cross- sectional longitudinal	To examine the associations between adherence to gender-typical behavior and substance use from adolescence to adulthood	*n* = 4617 males; *n* = 5660 females	Self-report number of occurrences (Waves 1 and 3) and days of cannabis use (Wave 4) in the past 30 days	Gender norms; gender typicality based on adherence to gender typical behaviours; behaviours included a range from individual actions (e.g., exercising) to states of being (e.g., getting sad) that correlated with being female or male.	Greater male typicality at wave one was associated with greater odds of high frequency cannabis and cigarette use and increased risk of use of one or more substances at Wave three (during emerging adulthood). Among females, there was a lower change in high frequency use and polysubstance use over time.

## Appendix A. Database Search Strategies

Search (1) August 2017

1	“gender transformative”. ti,ab.
2	(“gender informed” or “gender integrated” or “gender responsive”). ti,ab.
3	(“sex informed” or “sex integrated” or “sex responsive”). ti,ab.
4	(“gender equalit *” or “gender equit *” or “gender inequality *” or “gender inequit *”). ti,ab.
5	(“sex equalit *” or “sex equit *” or “sex inequality *” or “sex inequit *”). ti,ab.
6	(“gender related” or “gender difference *” or “gender disparit *”). ti,ab.
7	(“sex related” or “sex difference*” or “sex disparit*”).ti,ab.
8	“gender comparison *”. ti,ab.
9	“sex comparison *”. ti,ab.
10	“compar* gender *”. ti,ab.
11	“compar * sex *”. ti,ab.
12	“gender based”.ti,ab.
13	“sex based”.ti,ab.
14	(“gender divers *” or “gender minorit *”). ti,ab.
15	“gender analys *”. ti,ab.
16	“sex analys *”. ti,ab.
17	(transgender * or “trans gender *” or LGBQT or LGBTQ or LGBT or LGB or lesbian * or gay or bisexual * or queer *). ti,ab.
18	(“transsexual *” or “trans sexual *”).ti,ab.
19	17 or 18
20	(transgender * or “trans gender *” or LGBQT or LGBTQ or LGBT or LGB or lesbian * or gay or bisexual * or queer * or “transsexual *” or “trans sexual *”). ti,ab.
21	(“non binary *” or nonbinar *). ti,ab.
22	Homosex *. ti,ab.
23	(“woman focused” or “woman focussed” or “girl focused” or “girl focussed” or “woman centred” or “girl centred” or “woman centered” or “girl centered” or “female focused” or “female focussed” or “female centred” or “female centered”). ti,ab.
24	(“man focused” or “man focussed” or “boy focused” or “boy focussed” or “man centred” or “boy centred” or “man centered” or “boy centered” or “male focused” or “male focussed” or “male centred” or “male centered”). ti,ab.
25	Transgender Persons/
26	Sexual Minorities/
27	Transsexualism/
28	Bisexuality/
29	exp Homosexuality/
30	Gender Identity/
31	(bigender * or “bi gender *”). ti,ab.
32	(“gender identit *” or “gender incongru *”). ti,ab.
33	“differently gendered”. ti,ab.
34	or/1–33 [GENDER]
35	exp Opioid-Related Disorders/
36	exp Analgesics, Opioid/
37	(opioid * or opiate *). ti,ab.
38	(fentanyl or phentanyl or Fentanest or Sublimaze or Duragesic or Durogesic or Fentora or “R 4263” or R4263). ti,ab.
39	(oxycontin or oxycodone or oxycodan or percocet or percodan). ti,ab.
40	(heroin or morphine). ti,ab.
41	or/36–40 [OPIOIDS]
42	Prescription Drug Misuse/ or Prescription Drug Overuse/
43	((“prescription drug” or “prescription drugs” or “prescribed drug” or “prescribed drugs”) and (dependen * or misuse * or mis-use * or abuse * or overuse * or over-use * or addict *)). ti,ab.
44	exp Substance-Related Disorders/
45	(“substance disorder *” or “substance related disorder *” or “substance use disorder *” or “drug use disorder *” or “drug related disorder *”). ti,ab.
46	(“over prescription” or “over prescribed”). ti,ab.
47	Drug Overdose/ or (overdose* or over-dose *).ti,ab.
48	or/42–47
49	35 or (41 and 48)
50	exp Alcohol-Related Disorders/
51	exp Alcohol Drinking/
52	(binge drink * or underage drink * or under-age drink * or problem drink * or heavy drink * or harmful drink * or alcoholi* or inebriat * or intoxicat *). ti,ab.
53	(“alcohol dependen *” or “alcohol misuse *” or “alcohol mis-use *” or “alcohol abuse *” or “alcohol overuse *” or “alcohol over-use *” or “alcohol addict *”). ti,ab.
54	alcohol. ti,ab. and (44 or 45)
55	Alcohol Abstinence/
56	or/50–55
57	“Tobacco Use Disorder”/
58	Tobacco/
59	Nicotine/
60	exp Tobacco Products/
61	exp “Tobacco Use”/
62	((cigar * or e-cigar * or tobacco or nicotine or smoking or vaping) and (dependenc * or misuse * or mis-use * or abuse * or overuse * or over-use * or addiction *)). ti,ab.
63	(58 or 59 or 60 or 61) and (44 or 45)
64	exp “Tobacco Use Cessation”/
65	exp “Tobacco Use Cessation Products”/
66	((tobacco or smoking) and cessation). ti,ab.
67	or/57,62–66
68	Marijuana Abuse/
69	Cannabis/
70	Marijuana Smoking/
71	exp Cannabinoids/
72	(marijuana or marihuana or hashish or ganja or bhang or hemp or cannabis or cannabinoid * or cannabidiol or tetrahydrocannabinol). ti,ab.
73	(69 or 70 or 71 or 72) and (43 or 44 or 45)
74	or/68,73
75	or/49,56,67,74
76	Harm Reduction/
77	(“harm reduction” or “reducing harm” or “reducing harmful” or “harm minimization” or “minimizing harm” or “minimizing harmful” or “harm minimisation” or “minimising harm” or “minimising harmful”). ti,ab.
78	exp Risk Reduction Behavior/
79	(“risk reduction” or “reducing risk” or “reducing risks” or “risk minimization” or “minimizing risk” or “minimizing risks” or “risk minimisation” or “minimising risk” or “minimising risks”). ti,ab.
80	or/76–79
81	exp Health Promotion/
82	(“health promotion” or “promoting health” or “promoting healthy” or “promoting wellness” or “patient education” or “consumer education” or “client education” or outreach or “wellness program” or “wellness programs” or “wellness programme” or “wellness programmes”). ti,ab.
83	81 or 82
84	Preventive Health Services/
85	Consumer Health Information/ or Health Literacy/
86	Secondary Prevention/
87	(prevention or “preventive health” or “preventive healthcare”). ti,ab.
88	or/84-87
89	(prevention or preventive). ti,ab.
90	88 or 89
91	Rehabilitation/
92	(abstain * or abstinence or detox * or rehab * or sobriety or sober or temperance or intervention * or cessation or recovery). ti,ab.
93	Methadone/tu [Therapeutic Use]
94	“methadone maintenance”. ti,ab.
95	Opiate Substitution Treatment/
96	(“opiate substitution” or “opioid substitution” or “withdrawal management” or “managing withdrawal”). ti,ab.
97	(treatment* or treating or therapy or therapies). ti,ab.
98	Intervention *. ti,ab.
99	or/91–98
100	or/80,83,88,99
101	or/80,83,90,99
102	34 and 75 and 101
103	limit 102 to (english language and yr = “2007–2017”) [Limit not valid in Cochrane Database of Systematic Reviews (CDSR); records were retained]
104	(Animals/ or Animal Experimentation/ or “Models, Animal”/ or (animal * or nonhuman * or non human * or rat or rats or mouse or mice or rabbit or rabbit or pig or pigs or porcine or dog or dogs or hamster or hamsters or fish or chicken or chickens or sheep or cat or cats or raccoon or raccoons or rodent * or horse or horses or racehorse or racehorses or beagle *). ti,ab.) not (Humans/ or (human * or participant * or patient or patients or child * or seniors or adult or adults). ti,ab.)
105	103 not 104
106	(editorial or comment or letter or newspaper article). pt.
107	105 not 106
108	(conference or conference abstract or conference paper or “conference review” or congresses). pt.
109	107 not 108EBM Reviews- Cochrane Database of Systematic Reviews < 2005 to 2 August 2017 >Embase < 1980 to 3 August 2017 >Ovid MEDLINE(R) Epub Ahead of Print, In-Process and Other Non-Indexed Citations, Ovid MEDLINE(R) Daily and Ovid MEDLINE(R) < 1946 to Present >EBM Reviews- Cochrane Central Register of Controlled Trials < July 2017 >
110	remove duplicates from 109EBM Reviews- Cochrane Database of Systematic Reviews < 2005 to 2 August 2017 >Embase < 1980 to 3 August 2017 >Ovid MEDLINE(R) Epub Ahead of Print, In-Process and Other Non-Indexed Citations, Ovid MEDLINE(R) Daily and Ovid MEDLINE(R) < 1946 to Present>EBM Reviews- Cochrane Central Register of Controlled Trials < July 2017 >
111	110 use ppez [MEDLINE]
112	110 use emezd [EMBASE]
113	110 not (111 or 112) [selected 2 only as 13 were conference abstracts]

Search 2: September 2017

After reviewing the returns from the original search in August 2017, we amended the search in September 2017 to identify studies on the health effects of substance use (for cannabis, alcohol, opioids, tobacco/nicotine). In addition, we added sex/gender terms and substance-specific terms. The search was amended as follows:

1. Health effects terms were added to the search terms.

(“health effect” or “heath effects” or “effect on health” or “effects on health” or “affect * health” or “affect * the health” or “heath impact *” or “impact * on health” or “impact * health”). ti,ab. [HEALTH EFFECTS]

These terms were searched in combination with the gender terms and substance terms as follows:

Concept 1—Gender/sex

AND

Concept 2—Substances (opioids, alcohol, tobacco, cannabis)

AND

Concept 3—health effects

1. “gender determinant*” or “gender specific” were added to the gender/sex terms (see lines 1–33 in original search strategy)
2. “alcohol use” or “use of alcohol” and “risky drink” were added to the alcohol terms

Search 3: April 2018

After identifying multiple papers relevant to our review that were not being captured by the original searches, we conducted a third search in April 2018. Based on analysis of the keywords in the articles that were missed, we amended the search as follows:

1. The following terms were added to the gender/sex terms:
  (woman or man or women or men or girl or boy or girls or boys or trans or transgender or transgendered or female or male or sex or gender). ti. [GENDER IN TI]
  A search was then conducted of the article titles only, combining the following concepts:
  Search strategy:
  Concept 1—Gender/sex terms
  AND
  Concept 2—Substances (opioids, alcohol, tobacco, cannabis) terms
  AND
  Concept 3—Harm reduction, health promotion, prevention, treatment, health effects terms
2. “heat not burn” was added to the tobacco terms.
3. The search included studies published up until April 2018

## Appendix B. Final Inclusion Criteria

*Study Design:*

- randomised-controlled trials (RCTs) (not already covered in an included systematic review)
- case-control studies
- interrupted time series
- cohort studies
- cross sectional studies
- observational studies
- systematic reviews
- qualitative studies
- grey literature sources
- case series

Note:

- Narrative reviews will not be included but saved as context.
- Case studies will be excluded.

The following types of literature will be included in the grey literature review:

- book chapters
- reports
- practice guidelines
- health policy documents
- unpublished research, theses

Note: magazines and books will be excluded from the grey literature.

*Countries of studies:*

- Australia, Austria, Belgium, Canada, Denmark, Finland, France, Germany, Greece, Iceland, Ireland, Italy, Luxembourg, Netherlands, New Zealand, Norway, Portugal, Spain, Sweden, Switzerland, United Kingdom, United States
- Studies published in all other countries will be excluded, including animal studies.
- Studies including data from multiple countries, that include an out of scope country, will be excluded if the data is not disaggregated.
- Systematic reviews which include studies from multiple countries will be included if reporting on one or more studies published in an eligible country.

*Date of publication:*

- The literature search will cover studies published between 2007 to 2017

*Language:*

- Only studies published in the English language will be included.

**Research Q1: PICO (Population, Intervention, Comparator, Outcome)**

Q(1) *How* do sex- and gender-related factors impact:

(a) patterns of use;
(b) health effects of;
(c) and prevention/treatment/or harm reduction outcomes for opioid, alcohol, tobacco and cannabis use?

*Population:*

- Women, girls, men, boys, trans people/ gender diverse people
  - All ages, demographics within the defined populations
- Studies that are conducted primarily with pregnant girls and women will be excluded.
- Studies addressing the fetal health effects of maternal/ paternal substance use will be excluded.
- Studies addressing the health effects of substance use on the infant among women who are breastfeeding will be excluded.
- Studies comparing heterosexual populations to LGBT populations, without sex or gender disaggregation will be excluded.

*Intervention:*

Q1 (a) and (b) includes non-intervention studies (e.g., patterns of use, health effects):

- Inclusive of tobacco in general (include e-cigarettes)
- Inclusive of all alcohol use (not just binge drinking)
- Inclusive of all opioid use issues (include illicit use/heroin, prescription opioids, etc.)
  ▪ Opioid use for cancer pain management will be excluded
- Inclusive of all purposes (therapeutic and recreational), forms and modes of ingestion of cannabis (e.g., smoking, vaping, edibles, extracts, etc.).
- Studies that report on “substance use” but do not disaggregate results by one or more of the four substances in our review will be excluded.

Q1 (c) Harm reduction, health promotion, prevention, treatment (including brief intervention) responses to opioids, alcohol, cannabis, tobacco/e-cigarettes

- Studies that report on “substance use” but do not disaggregate results by one or more of the four substances in our review will be excluded.
- Opioid substitution therapy for substances other than opioids (e.g., cocaine, methamphetamine) will be excluded

*Comparator:*

- Many Q1 (a) and (b) studies will be descriptive/ prevalence studies (not intervention studies) and may not include a comparator.
- Many qualitative and grey literature sources will likely not include comparators
- Q1 (c) studies *must* include a comparison between gender groups e.g., women vs. men; sub-groups of women/ men OR if sex- or gender- based factors are described or discussed in the study (e.g., masculinity norms, hormones etc.). Q1c studies that do not compare gender groups or describe sex- or gender-based factors will be excluded.

*Outcome:*

- For non-intervention studies: prevalence/patterns of use (frequency of use, form and method of ingestion, etc.);
- For intervention studies (Q1c): outcomes reported in the reviews will be the outcomes that are reported in the individual papers that are reviewed. Relevant outcomes from the included studies might include:
  - Changes in substance use (uptake/initiation, harms associated with use cessation, reduction)
  - Changes in client perceptions/attitudinal change
  - Changes in service provider perceptions
  - Changes in retention/treatment completion
  - Increased use of services
  - improved health and quality of life outcomes

Note: Studies that report on one or more of the four substances in relation to sex/gender *only* in the baseline characteristics of the sample will be excluded, even if statistical significance is reported.

**Research Q2: PICO (Population, Intervention, Comparator, Outcome)**

(Q2) *What* harm reduction, health promotion/prevention and treatment interventions and programs are available *that include sex, gender and gender transformative elements* and how effective are these in addressing opioid, alcohol, tobacco and cannabis use?

*Population:*

- Women, girls, men, boys, trans people/gender-diverse people
  - All ages, demographics within the defined populations
- Studies that are conducted primarily with pregnant girls and women will be excluded.
- Studies addressing the fetal health effects of maternal/paternal substance use will be excluded.
- Studies addressing the health effects of substance use on the infant among women who are breastfeeding will be excluded.

*Intervention:*

- Harm reduction, health promotion, prevention, treatment (including brief intervention) responses to opioids, alcohol, cannabis, tobacco/e-cigarettes including some *sex, gender and/or gender transformative elements*
  - Studies that report on ‘substance use’ will be included if they potentially contain one of the four substances. However, if substance use is defined, and does not contain alcohol, tobacco, opioids or cannabis it will be excluded.
  - Opioid substitution therapy for substances other than opioids (e.g., cocaine, methamphetamine) will be excluded.
- Examples of sex specific elements (address biological differences in substance use and dependence):
  - administering different types or quantities of pharmacotherapies based on evidence of biological differences in drug metabolism/effectiveness
  - timing tobacco-cessation intervention for young women based on the menstrual cycle (hormonal fluctuations impact withdrawal)
- Examples of possible gender/gender-transformative elements:
  - address gender-based violence
  - provide social support
  - address caregiving
  - address poverty
  - address negative gender stereotypes
  - include education or messaging on gender norms/relations
  - address employment issues/work-related stress
  - address discrimination and violence related to gender identity

Interventions to address these four substances are:

- Inclusive of tobacco in general (include e-cigarettes)
- Inclusive of all alcohol use (not just binge drinking)
- Inclusive of all opioid use issues (include illicit use/heroin, prescription opioids, etc.)
  ▪ Opioid use for cancer pain management will be excluded
- Inclusive of all purposes (therapeutic and recreational), forms and modes of ingestion of cannabis (e.g., smoking, vaping, edibles, extracts, etc).

Note: Methadone maintenance therapy will only be included if it is provided to opioid users (i.e., exclude if provided to treat substances outside of scope such as cocaine).

*Comparator:*

- No intervention or usual practice (i.e., interventions that are not gender-informed/gender-transformative, sex-specific), or the comparison of two intervention types.
  - Many qualitative and grey literature sources will likely not include comparators.

*Outcome:*

- Outcomes reported in the reviews will be the outcomes that are reported in the individual papers that are reviewed. Relevant outcomes from the included studies might include:
  - Changes in substance use (uptake/initiation, harms associated with use, cessation, reduction)
  - Changes in client perceptions/attitudinal change
  - Changes in service provider perceptions
  - Changes in retention/treatment completion
  - Increased use of services
  - improved health and quality of life outcomes
  - changes in health and gender equity

Note: Studies that report on one or more of the four substances in relation to sex/gender *only* in the baseline characteristics of the sample will be excluded, even if statistical significance is reported.
